# Prognostic values of the mRNA expression of natural killer receptor ligands and their association with clinicopathological features in breast cancer patients

**DOI:** 10.18632/oncotarget.25506

**Published:** 2018-06-05

**Authors:** Ali Abouelghar, Reem Hasnah, Ghina Taouk, Mohamad Saad, Manale Karam

**Affiliations:** ^1^ Cancer Research Center, Qatar Biomedical Research Institute, Hamad Bin Khalifa University, Qatar Foundation, Doha, Qatar; ^2^ Department of Biological Sciences, Carnegie Mellon University in Qatar, Doha, Qatar; ^3^ Cancer Research Center, Qatar Biomedical Research Institute, Hamad Bin Khalifa University, Qatar Foundation, Doha, Qatar; ^4^ Qatar Computing Research Institute, Hamad Bin Khalifa University, Doha, Qatar; ^5^ Cancer Research Center, Qatar Biomedical Research Institute, Hamad Bin Khalifa University, Qatar Foundation, Doha, Qatar

**Keywords:** natural killer cells, breast cancer, NK receptor ligands, Kaplan-Meier plotter, prognosis

## Abstract

**Background:**

Natural killer (NK) cells are lymphocytes of the innate immune system that have potent cytotoxic activity against tumor cells. NK cell recognition and activity towards cancer cells are regulated by an integrated interplay between numerous inhibitory and activating receptors acting in concert to eliminate tumor cells expressing cognate ligands. Despite strong evidence supporting the role of NK cells in breast cancer (BC) control, BC still develops and progresses to form large tumors and metastases. A major mechanism of BC escape from NK immunity is the alteration of the expression of NK receptor ligands. The aim of this study was to determine whether NK receptor ligands’ mRNA expression might influence prognosis in BC patients and whether these effects differ by molecular subtypes and clinicopathological features.

**Methods:**

We used the KM plotter platform to analyze the correlation between mRNA expression of 32 NK receptor ligands and relapse-free survival (RFS) and overall survival (OS) in 3951 and 1402 BC patients, respectively. The association with tumor subtypes and clinicopathological features was determined. BC samples were split into high and low expression groups according to the best cutoff value and the two patient cohorts were compared by Kaplan–Meier survival plots. The hazard ratios with 95% confidence intervals and log rank *P* values were calculated and FDR-adjusted for multiple testing correction. The data was considered to be statistically significant when FDR-adjusted *P* value < 0.05.

**Results:**

High mRNA expression of around 80% of ligands for NK activating and inhibitory receptors associated with better RFS, which correlated with longer OS for only about half of the NK-activating ligands but for most NK-inhibitory ligands. Also, five NK-activating ligands correlated with worse prognosis. These prognostic values were differentially associated with the BC clinical criteria. In addition, the favorable prognostic influence of NK-activating ligands’ upregulation, as a whole, was mainly significantly associated with HER2-positive and basal-like subtypes, lymph node positive phenotype, and high-grade tumors.

**Conclusions:**

NK receptor ligands appear to play an important role in defining BC patient prognosis. Identification of a group of patients with worse prognosis expressing high levels of NK-activating ligands and low levels of NK-inhibitory ligands makes them ideal potential candidates for NK-based immunotherapy to eliminate residual tumor cells, prevent relapse and improve patient survival.

## INTRODUCTION

Breast cancer (BC) treatment has experienced several changes in the past decades due to the discovery of specific prognostic and predictive biomarkers that allowed its classification and enabled the application of more individualized therapies to the different molecular subgroups [1–4]. Among these biomarkers, steroid hormone receptors such as estrogen receptor (ER) and progesterone receptor (PR) in concert with the oncogene ErbB-2/human epidermal growth factor receptor 2 (HER-2) are critical determinants of the four main molecular subtypes of BC. Tumors of luminal A and luminal B subtypes are hormone receptor-positive (ER and/or PR-positive) and represent around 70% of all BCs [5]. Luminal A tumors are often low grade with slow tumor growth and have the best prognosis. Luminal B cancers generally grow slightly faster than luminal A cancers and have a slightly worse prognosis. Both luminal types are treated with endocrine therapy [5]. On the other hand, HER2-positive subtypes (around 15% of BCs) overexpress HER2, tend to grow faster than luminal cancers and can have a worse prognosis. But, they are often successfully treated with HER2-targeted therapies [6]. Triple negative (also called basal-like) tumors are ER negative, PR negative, and HER-2 negative. Although the basal-like subtype is only found in about 15% of BC diagnoses, it has been shown to be aggressive, unresponsive to treatment and, ultimately, indicative of a poor prognosis [7–11]. These classical molecular biomarkers (i.e. ER, PR, and HER2) are generally complemented with traditional clinicopathological factors (including tumor grade, lymph-node metastases and p53 status) and conventionally used for patient prognosis and management [12]. Recently, with the introduction of high-throughput technologies, numerous multigene tests such as urokinase plasminogen activator (uPA)-PAI-1, Oncotype DX, MammaPrint, EndoPredict, Breast Cancer Index (BCI) and Prosigna (PAM50), may be performed in specific subgroups of BC patients to predict outcome and aid adjunct therapy decision-making [2]. Current prospective clinical trials are seeking evidence for their definitive role in BC. The advances in molecular biomarkers and the progress in treatment modalities have together contributed to improvements in overall survival of BC patients. However, in many cases tumors do not respond to the currently available treatments or relapse after initial response [13]. Therefore, new biomarkers are needed to quantify the residual risk of BC patients and to indicate the potential value of additional treatment strategies to eliminate these resistant tumors.

Recent major scientific advances have demonstrated the importance of the immune system in malignant diseases including BC. Both innate and adaptive immune cells actively prevent neoplastic development in a process called ‘cancer immunosurveillance’ [14–16]. However, due to their genetic instability, malignant cells can develop several mechanisms to evade immunosurveillance [17, 18]. Therefore, strategies designed to harness the immune system are the focus of several recent promising therapeutic approaches for cancer patients [19, 20]. Natural killer (NK) cells are lymphocytes of the innate immune system that play a critical role in host immune responses against tumor growth and metastasis [21–23]. Following the progress in NK cell biology field and in understanding NK function, these lymphocytes have recently become a powerful cancer immunotherapy tool that presents several advantages [24]. Furthermore, a significant piece of experimental and clinical evidence supports the role of NK cells in BC control [22, 25–33], suggesting that NK cell-based therapy may become a potent strategy for the eradication of residual BC cells, prevention of relapse and improvement of patient survival.

NK cells recognize their target through a complex array of regulatory receptors that monitor cell surfaces of autologous or host cells for an aberrant expression of major histocompatibility complex (MHC) class I molecules and cell stress markers, which frequently occur in cancer cells [34, 35]. In fact, upon cellular transformation, MHC class I expression on the cell surface is often reduced or lost to evade recognition by antitumor T cells. When NK cells encounter transformed cells lacking MHC class I, their inhibitory receptors are not engaged, and the unsuppressed activating signals, in turn, can trigger cytokine secretion and targeted attack of the transformed cells [36, 37]. In parallel, cellular stress and DNA damage (occurring in malignant transformation) result in upregulation of “stress ligands” that can be recognized by activating NK receptors [38]. Thus, human tumor cells that have lost self-MHC class I expression or bear “altered-self ” stress-inducible proteins are ideal targets for NK recognition and cytotoxicity [36, 39, 40]. However, during cancer progression, tumor cells deregulate the expression of these ligands by several mechanisms in order to escape from NK detection and elimination [41–48]. Thus, analysis of the expression of NK receptor ligands in BCs may allow the determination of new biomarkers to quantify the residual risk of patients and to indicate the potential value of additional NK-based treatment strategies.

The “Kaplan–Meier plotter” (KM plotter) is an online platform (http://kmplot.com/analysis/) that can be used to assess the effect of 54,675 genes on patient survival using 10,461 cancer samples (including breast, ovarian, lung and gastric cancers). This platform is established by using gene expression data and patient survival information downloaded from Gene Expression Omnibus (GEO) (Affymetrix microarrays only), European Genome-phenome Archive (EGA), and The Cancer Genome Atlas (TCGA). The database is handled by a PostgreSQL server, which integrates gene expression and clinical data simultaneously. The KM plotter was validated and widely used in many studies to identify a number of genes, as prognostic markers or potential drug targets in BC [49–54], lung cancer [51, 55–57], ovarian cancer [58–60] and gastric cancer [51, 61–63].

The aim of the present study was to determine the prognostic roles of mRNA expression of NK receptor ligands in BC patients and their association with different BC molecular subtypes and clinicopathological features. Therefore, we performed systematic literature screening to select and ascertain all NK-regulatory ligands for NK receptors identified to date. Then, we utilized to KM plotter to analyze their effect on BC patient relapse-free and overall survivals.

## RESULTS

### Prognostic values of the mRNA expression of NK receptor ligands in BC patients

First, we systematically screened the literature to identify ligands for NK receptors that regulate NK activity and cytotoxicity towards the target cells that express these ligands. In total, we selected 39 NK-regulatory ligands for NK receptors [64–133] (Table 1). Among these, 27 ligands for 17 NK receptors that induce the cytotoxic activity of NK cells towards the target cells (NK-activating ligands), 8 ligands for 8 NK receptors whose interaction inhibits the NK activity (NK-inhibitory ligands) and 4 ligands that can bind different NK receptors to either activate or inhibit NK activity (NK-activating and inhibitory ligands).

**Table 1:** Activating and Inhibitory ligands for NK receptors

Ligand	Corresponding NK receptor(s)	References	Probe ID
NK-activating ligands			
AICL	NKp80	[76]	209732_at
B7-1, CD80	Unknown	[68, 71, 77]	1554519_at
B7-2, CD86	Unknown	[71, 77]	205685_at
B7-H6	NCR3, NKp30	[66]	N/A
BAT3	NCR3, NKp30	[73]	210208_x_at
CD27	CD70	[64]	206150_at
CD48	2B4, CD244	[67, 69, 70]	204118_at
CD58	CD2	[132, 133]	205173_x_at
CD70	CD27	[74]	206508_at
CD72	CD100	[72]	215925_s_at
Fcγ fragment of IgG	Fcγ receptor III, CD16	[75]	N/A
Heparan sulfate	1. NCR1, NKp46 2. NCR2, NKp44 3. NCR3, NKp30	1. [65, 88] 2. [88] 3. [65, 88]	N/A
KMT2E, NKp44L	NCR2, NKp44	[80]	226100_at
MICA	NKG2D	[79]	205904_at
MICB	NKG2D	[79, 83]	206247_at
NECL2	CRTAM	[81]	209031_at
SLAMF6, NTB-A	SLAMF6, NTB-A	[85]	1552497_a_at
SLAMF7, CS1	SLAMF7, CS1	[91]	222838_at
TNFSF9, 4-1BBL, CD137L	TNFRSF9, 4-1BB, CD137	[86, 89, 90, 93]	206907_at
ULBP1	NKG2D	[83]	221323_at
ULBP2	NKG2D	[83]	238542_at
ULBP3	NKG2D	[83]	231748_at
ULBP4	NKG2D	[78, 82]	1552777_a_at
ULBP5	NKG2D	[78]	N/A
ULBP6	NKG2D	[84]	N/A
VIM	NCR1, NKp46	[87]	201426_s_at
Viral HA, HN	1. NCR1, NKp46 2. NCR2, NKp44	1. [92] 2. [94]	N/A
**NK-activating and inhibitory ligands**			
HLA-C	1. CD160 (activating) 2. KIR2DS1, p50.1 (activating) 3. KIR2DS4, NKAT8 (activating) 4. KIR2DL1, p58.1 (inhibitory) 5. KIR2DL2, p58.2 (inhibitory) 6. KIR2DL3, p58 (inhibitory)	1. [104] 2. [95, 107] 3. [97, 103] 4.5.6. [100, 108, 109]	216526_x_at
HLA-E	1. NKG2A (inhibitory) 2. NKG2B (inhibitory) 3. NKG2C (activating) 4. NKG2E (activating)	1. [96, 98, 101, 102, 105, 106] 2. [96,98] 3. [96, 101, 102] 4. [102]	200904_at
NECL5, PVR	1. CD96 (activating) 2. CD226, DNAM1 (activating) 3. TIGIT (inhibitory)	1. [99, 115, 123] 2. [112] 3. [124]	214443_at
NECTIN2, CD112, PVRL2	1. CD226, DNAM1 (activating) 2. PVRIG, CD112R (inhibitory) 3. TIGIT (inhibitory)	1. [112] 2. [125] 3. [124, 125]	203149_at
**NK-inhibitory ligands**			
CEACAM1	CEACAM1	[118, 119]	209498_at
CLEC2D, LLT1	CD161, NKRP1A	[110, 122]	235522_at
COL3A1	LAIR1, CD305	[117, 120]	211161_s_at
hCMV PP65	NCR3, NKp30	[111]	N/A
HLA-A	1. KIR3DL2, p140 2. LILRB1, ILT2	1. [114, 116, 121, 126] 2. [113]	215313_x_at
HLA-B	1. KIR3DL1, NKB1 2. LILRB1, ILT2	1. [128] 2. [113]	209140_x_at
PDL1	PD-1	[127, 130]	227458_at
PDL2	PD-1	[127, 129]	220049_s_at

Abbreviations: AICL, activation-induced C-type lectin; B7-1/2, B-lymphocyte activation antigen B7-1/2; B7-H6, B7 homolog 6; BAT3, HLA-B-associated transcript 3; CD, cluster of differentiation; CEACAM1, carcinoembryonic antigen-related cell adhesion molecule 1; CLEC2D, C-type lectin domain family 2 member D; COL3A1, collagen type 3 alpha 1; CRTAM, class I-restricted T-cell-associated molecule; CS1, CD2 subset 1; DNAM1, DNAX accessory molecule-1; HA, haemagglutinin; hCMV PP65, human cytomegalovirus pp65; HLA-A/B/C/E, human leukocyte antigen-A/B/C/E; HN, haemagglutinin neuramidase; ILT2, Ig-like transcript 2; KIRxDLy, killer cell immunoglobulin like receptor “x” Ig domains and long cytoplasmic tail “y”; KLRG1, killer cell lectin like receptor G1; KMT2E, lysine methyltransferase 2E; LAIR1, leukocyte associated immunoglobulin like receptor 1; LILRB1, leukocyte immunoglobulin like receptor B1; LLT1, Lectin-Like Transcript-1; MICA/B, MHC class I polypeptide-related sequence A/B; NCR1/2/3, natural cytotoxicity triggering receptor 1/2/3; NECL2/5, nectin-like protein 2/5; NECTIN2, nectin cell adhesion molecule 2; NKG2A/B/C/D/E, natural-killer group 2 member A/B/C/D/E; NKRP1A, NK receptor-P1A; NTB-A, NK-, T-, and B-cell antigen; N/A, not available; PD-1/2, programmed death receptor-1/2; PDL1, programmed death ligand 1; PVR, poliovirus receptor; PVRIG, PVR related immunoglobulin domain containing); PVRL2, poliovirus receptor related 2; SLAMF6/7, signaling lymphocytic activation molecule family member 6/7; TIGIT, T-cell immunoreceptor with Ig and ITIM domains; TNFSF9, tumor necrosis factor superfamily member 9; ULBP1-6, UL16 binding protein 1-6; VIM, vimentin.

**Figure 1:**
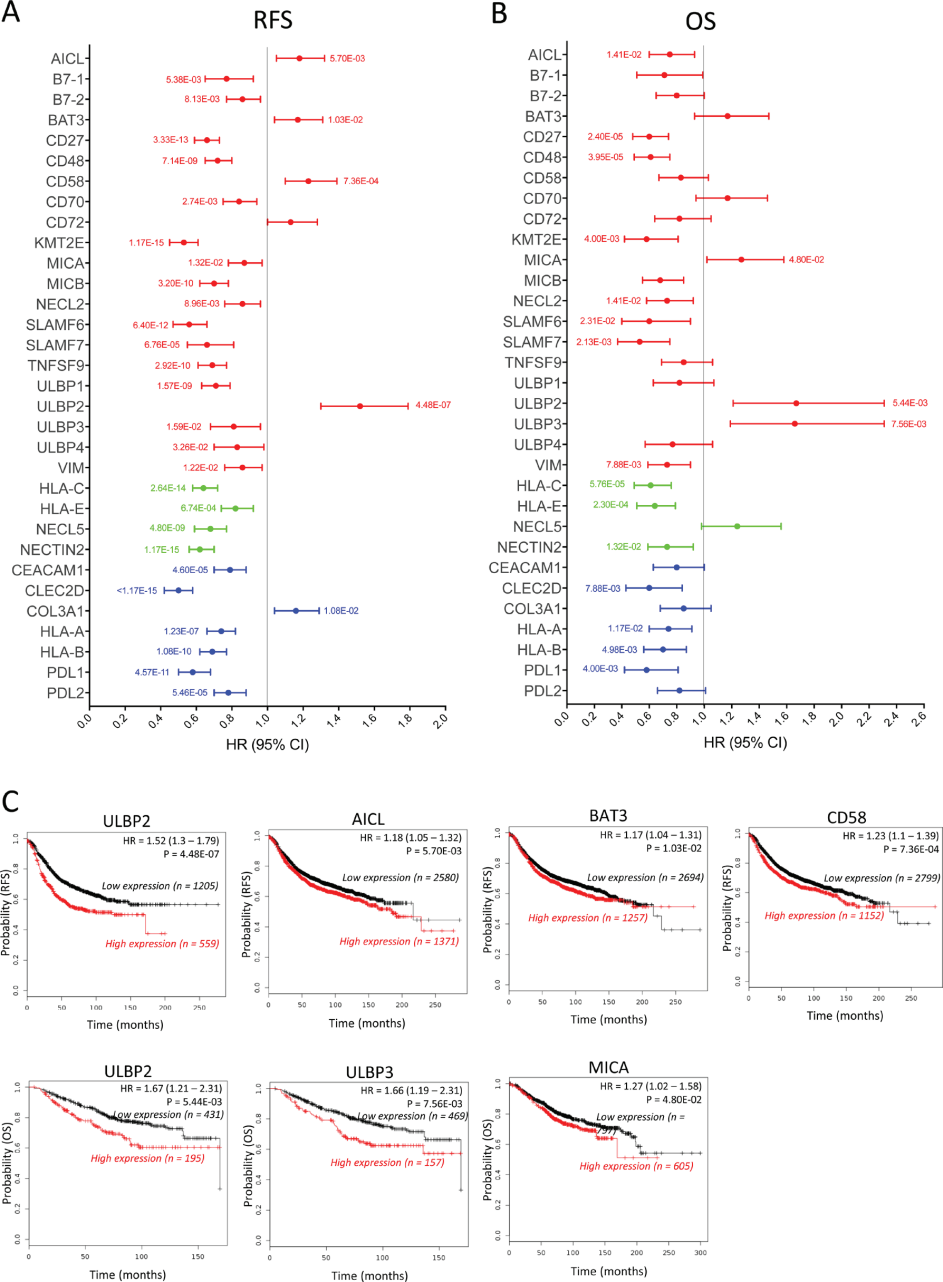
Prognostic values of the mRNA expression of NK receptor ligands in all breast cancer patients The correlation of the individual expression of 32 NK receptor ligands’ mRNA to RFS (**A**) and OS (**B**) was analyzed, in 3951 and 1402 breast cancer patients, respectively, on the KM plotter database. For each ligand, the bar represents the HR (95% CI). The *p* value is indicated next to each bar when the data is statistically significant (*p* value < 0.05). The bar color (red, blue or green) represents the effect of the ligand on NK activity (activation, inhibition or both depending on the receptor type, respectively). The line at HR = 1 separates the ligands according to the prognostic influence of their high mRNA expression; better survival (HR < 1) and worse survival (HR > 1). (**C**) Kaplan–Meier survival plots of RFS (upper plots) and OS (lower plots) durations in BC patients with the expression levels of the NK-activating ligands that specifically correlated with poor prognosis. The “n” values represent the number of BC patients in each cohort.

Next, we used the KM plotter platform to determine the influence of the mRNA expression level of 32 ligands from the identified genes (Table 1) on the survival of BC patients. The other 7 ligands (Table 1, Probe ID = N/A) could not be analyzed because they were not available on the KM plotter (viral genes, Fcγ fragment of IgG, heparan sulfate and three other genes that had no related probe set). The analysis results of the correlation between mRNA expression of the 21 NK-activating ligands for NK receptors and survival are presented in Figure 1A (Red) for RFS and Figure 1B (Red) for OS. Patients with high mRNA expression of the NK-activating ligands B7-1 (HR = 0.77, 95% CI = 0.65–0.92, *p* = 0.0053), B7-2 (HR = 0.86, 95% CI = 0.77–0.96, *p* = 0.0081), CD27 (HR = 0.66, 95% CI = 0.59–0.73, *p* = 0.00000000000033), CD48 (HR = 0.72, 95% CI = 0.65–0.8, *p* = 0.0000000071), CD70 (HR = 0.84, 95% CI = 0.75–0.94, *p* = 0.0027), KMT2E (HR = 0.53, 95% CI = 0.45–0.61, *p* = 0.0000000000000011), MICA (HR = 0.87, 95% CI = 0.78–0.97, *p* = 0.013), MICB (HR = 0.7, 95% CI = 0.62–0.78, *p* = 0.00000000032), NECL2 (HR = 0.86, 95% CI = 0.76–0.96, *p* = 0.0089), SLAMF6 (HR = 0.56, 95% CI = 0.47–0.66, *p* = 0.0000000000064), SLAMF7 (HR = 0.66, 95% CI = 0.55–0.81, *p* = 0.000067), TNFSF9 (HR = 0.69, 95% CI = 0.61–0.77, *p* = 0.00000000029), ULBP1 (HR = 0.71, 95% CI = 0.63–0.79, *p* = 0.0000000015), ULBP3 (HR = 0.81, 95% CI = 0.68–0.96, *p* = 0.015), ULBP4 (HR = 0.83, 95% CI = 0.7–0.98, *p* = 0.032) and VIM (HR = 0.86, 95% CI = 0.76–0.97, *p* = 0.012) had significantly longer RFS than patients with lower mRNA expression of these ligands (Figure 1A, Red). The longer RFS in the patients expressing high levels of the above-mentioned 16 NK-activating ligands significantly was associated with longer OS for only 7 ligands; CD27, CD48, KMT2E, NECL2, SLAMF6, SLAMF7 and VIM (Figure 1B, Red). However, despite the longer RFS, MICA (HR = 1.27, 95% CI = 1.02–1.58, *p* = 0.048) and ULBP3 (HR = 1.66, 95% CI = 1.19–2.31, *p* = 0.0075) were associated with shorter OS, while B7-1, B7-2, CD70, MICB, TNFS9, ULBP1 and ULBP4 didn’t significantly correlate with OS (Figure 1B, Red).

On the other hand, patients with high mRNA expression of the NK-activating ligand ULBP2 had significantly worse OS (HR = 1.67, 95% CI = 1.21–2.31, *p* = 0.0054) and RFS (HR = 1.52, 95% CI = 1.3–1.79, *p* = 0.00000044). In addition, patients with high mRNA expression of the NK-activating ligands BAT3 (HR = 1.17, 95% CI = 1.04–1.29, *p* = 0.01), AICL (HR = 1.18, 95% CI = 1.05–1.32, *p* = 0.0057) and CD58 (HR = 1.23, 95% CI = 1.1–1.39, *p* = 0.00073) had shorter RFS but not OS. CD72 didn’t show any significant effect on OS or RFS.

The correlation between mRNA expression of 7 NK-inhibitory ligands for NK receptors and survival (RFS and OS) in BC patients was also analyzed (Figure 1A and 1B, Blue). Except for COL3A1 (HR = 1.16, 95% CI = 1.04–1.21, *p* = 0.01) that associated with shorter RFS, patients with high mRNA expression of CEACAM1 (HR = 0.79, 95% CI = 0.7–0.88, *p* = 0.000046), CLEC2D (HR = 0.5, 95% CI = 0.42–0.58, *p* < 0.000000000000001), HLA-A (HR = 0.74, 95% CI = 0.66–0.82, *p* = 0.00000012), HLA-B (HR = 0.69, 95% CI = 0.62–0.77, *p* = 0.0000000001), PDL1 (HR = 0.58, 95% CI = 0.5–0.68, *p* = 0.000000000045) and PDL2 (HR = 0.78, 95% CI = 0.7–0.88, *p* = 0.000054) had significantly higher RFS than do patients with lower mRNA expression of these ligands (Figure 1A, Blue), which also associated with longer OS for CLEC2D, HLA-A, HLA-B and PDL1 (Figure 1B, Blue).

The four ligands that can bind activating and inhibitory NK receptors; HLA-C, HLA-E, NECL5 and NECTIN2 all associate with better RFS and OS, except for NECL5 that only significantly associated with better RFS (Figure 1A and 1B, Green).

Taken together, these results indicate that the high mRNA expression of most (around 80%) ligands for NK activating and inhibitory receptors associate with better RFS in BC patients. However, while the longer RFS correlates with longer OS for most NK-inhibitory ligands, in only about half of the NK-activating ligands it significantly correlates with longer OS. On the other hand, six NK-activating ligands (AICL, BAT3, CD58, MICA, ULBP2 and ULBP3) correlated with worse prognosis (shorter RFS and/or OS) in BC patients (Figure 1C).

### Association between the prognostic role of the mRNA expression of NK receptor ligands and the BC subtypes

**Figure 2:**
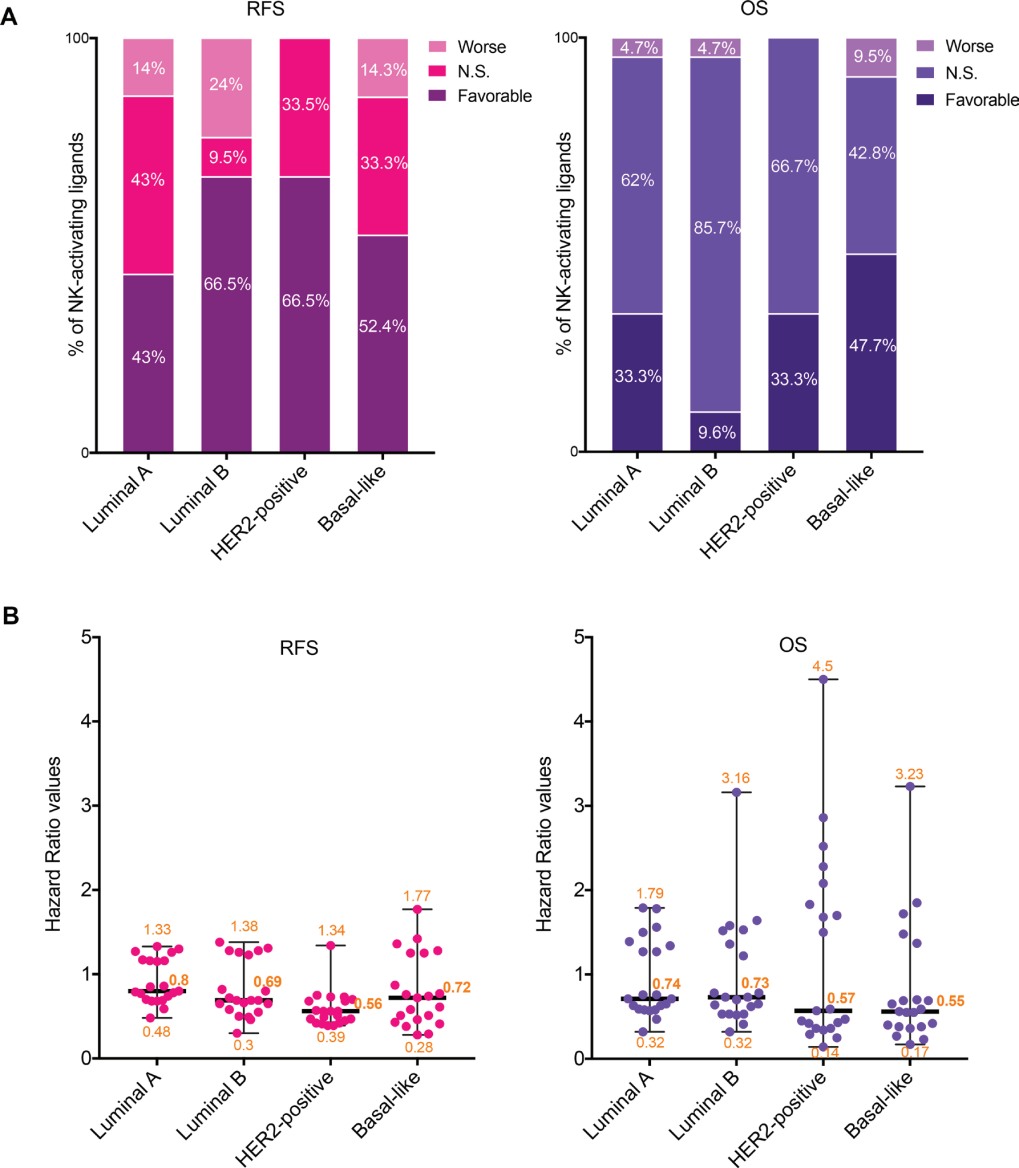
Prognostic values of the mRNA expression of NK-activating ligands depending on the BC molecular subtype (**A**) Percentages of the NK-activating ligands whose high mRNA expression significantly associated (Favorable and Worse) or not (N.S.) with RFS (Left graph) or OS (Right graph) in the different BC subtypes. “Favorable“ indicates the association of high mRNA expression with longer RFS/OS, representing good prognosis. “Worse” indicates the association of the high mRNA expression with shorter RFS/OS, representing bad prognosis. “N.S.” indicates no significant association with prognosis. (**B**) Individual value plots visualizing the distribution of the hazard ratio (HR) values of the NK-activating ligands in the different BC subtypes. The bars represent the median, lower and higher HR values for all ligands in each subtype.

In BC, the treatment selection and clinical outcome are mainly defined by molecular subtypes. Therefore, we next checked whether the prognostic influence of NK receptor ligands might be dependent on any specific BC subtype(s) (luminal A, luminal B, HER2-type and/or basal-like) (Table 2 and Figure 2).

**Table 2:** Association between the prognostic role of mRNA expression of NK receptor ligands and BC subtypes

		RFS				OS			
	Ligand	Luminal A	Luminal B	HER2-positive	Basal-like	Luminal A	Luminal B	HER2-positive	Basal-like
NK-activating ligands	AICL HR (95%CI) *p*-value	1.3 (1.09–1.56) **0.0098**	1.31 (1.05–1.62) **0.017**	0.73 (0.49–1.09) 0.14	1.25 (0.97–1.61) 0.092	0.71 (0.5–1.01) 0.11	0.73 (0.48–1.1) 0.17	0.34 (0.18–0.65) **0.0048**	0.56 (0.34–0.94) **0.046**
	B7-1 HR (95%CI) *p*-value	1.16 (0.9–1.48) 0.25	0.65 (0.48–0.89) **0.008**	0.51 (0.32–0.82) **0.008**	0.41 (0.29–0.57) **0.000000087**	1.5 (0.9–2.49) 0.16	0.78 (0.35–1.73) 0.54	0.42 (0.19–0.95) 0.056	0.23 (0.12–0.45) **0.000012**
	B7-2 HR (95%CI) *p*-value	1.16 (0.97–1.39) 0.11	0.72 (0.6–0.88) **0.0014**	0.56 (0.38–0.82) **0.0052**	0.46 (0.36–0.6) **0.0000000099**	1.39 (0.93–2.09) 0.16	0.73 (0.5–1.05) 0.13	0.45 (0.24–0.87) **0.041**	0.42 (0.25–0.69) **0.0011**
	BAT3 HR (95%CI) *p*-value	1.27 (1.06–1.52) **0.018**	1.38 (1.13–1.68) **0.0019**	0.56 (0.38–0.83) **0.0058**	0.87 (0.67–1.14) 0.32	1.79 (1.25–2.58) **0.014**	1.36 (0.85–2.17) 0.20	0.43 (0.2–0.92) 0.053	0.55 (0.3–1.01) 0.070
	CD27 HR (95%CI) *p*-value	0.69 (0.58–0.82) **0.00015**	0.55 (0.46–0.67) **0.000000051**	0.39 (0.26–0.57) **0.000016**	0.38 (0.3–0.5) **5.60E–13**	0.58 (0.4–0.84) **0.017**	0.53 (0.37–0.77) **0.014**	0.25 (0.12–0.54) **0.0017**	0.36 (0.22–0.58) **0.000076**
	CD48 HR (95%CI) *p*-value	0.86 (0.72–1.02) 0.091	0.58 (0.47–0.71) **0.0000009**	0.42 (0.28–0.62) **0.00012**	0.43 (0.33–0.55) **3.91E–11**	0.59 (0.41–0.86) **0.021**	0.52 (0.35–0.78) **0.014**	0.36 (0.18–0.69) **0.0074**	0.27 (0.16–0.44) **0.00000023**
	CD58 HR (95%CI) *p*-value	1.26 (1.04–1.52) **0.027**	1.28 (1.05–1.56) **0.016**	0.57 (0.39–0.85) **0.008**	1.42 (1.08–1.85) **0.015**	0.71 (0.48–1.03) 0.12	0.64 (0.42–0.99) 0.12	0.29 (0.14–0.6) **0.004**	0.65 (0.39–1.08) 0.11
	CD70 HR (95%CI) *p*-value	0.85 (0.71–1.01) 0.089	0.69 (0.57–0.84) **0.00032**	0.39 (0.24–0.65) **0.00071**	0.76 (0.57–1) 0.062	1.27 (0.89–1.83) 0.24	0.78 (0.53–1.12) 0.20	2.28 (1–5.21) 0.068	0.7 (0.42–1.18) 0.19
	CD72 HR (95%CI) *p*-value	1.17 (0.96–1.43) 0.11	0.65 (0.51–0.83) **0.00089**	0.49 (0.33–0.73) **0.00076**	0.51 (0.37–0.72) **0.00015**	1.56 (1.01–2.4) 0.1	0.65 (0.4–1.06) 0.13	0.47 (0.24–0.92) 0.053	0.4 (0.23–0.71) **0.0032**
	KMT2E HR (95%CI) *p*-value	0.48 (0.37– 0.61) **0.000000036**	0.46 (0.34–0.64) **0.0000044**	0.56 (0.33–0.95) **0.04**	0.74 (0.54–1.03) 0.086	0.32 (0.19–0.54) **0.00016**	0.62 (0.31–1.21) 0.17	4.5 (1.06–19.14) 0.053	0.69 (0.34–1.43) 0.32
	MICA HR (95%CI) *p*-value	0.77 (0.64–0.93) **0.012**	1.23 (1.01–1.49) **0.043**	0.75 (0.49–1.14) 0.18	1.28 (0.97–1.69) 0.086	1.27 (0.87–1.86) 0.24	1.64 (1.13–2.39) 0.064	1.68 (0.88–3.21) 0.14	1.85 (1.13–3.03) **0.025**
	MICB HR (95%CI) *p*-value	0.74 (0.62–0.89) **0.0021**	0.69 (0.57–0.84) **0.00038**	0.47 (0.31–0.7) **0.00064**	0.51 (0.4–0.66) **0.00000045**	0.76 (0.53–1.1) 0.20	0.7 (0.48–1.02) 0.13	0.57 (0.3–1.1) 0.12	0.38 (0.23–0.63) **0.00025**
	NECL2 HR (95%CI) *p*-value	0.7 (0.59–0.83) **0.00016**	1.28 (1.03–1.59) **0.029**	1.34 (0.89–2.03) 0.17	1.36 (1.04–1.77) **0.033**	0.47 (0.33–0.67) **0.00036**	1.58 (1.01–2.49) 0.12	1.83 (0.96–3.52) 0.093	1.37 (0.82–2.29) 0.23
	SLAMF6 HR (95%CI) *p*-value	0.59 (0.45–0.77) **0.00022**	0.3 (0.19–0.48) **0.00000075**	0.42 (0.26–0.7) **0.0011**	0.28 (0.2–0.39) **1.42E–14**	0.65 (0.38–1.11) 0.16	0.32 (0.12–0.82) 0.076	0.59 (0.22–1.59) 0.29	0.38 (0.2–0.73) **0.0059**
	SLAMF7 HR (95%CI) *p*-value	0.8 (0.61–1.05) 0.11	0.5 (0.33–0.74) **0.00075**	0.41 (0.26–0.65) **0.00057**	0.29 (0.21–0.4) **1.03E–13**	0.76 (0.45–1.27) 0.33	0.41 (0.19–0.88) 0.077	0.14 (0.03–0.61) **0.0092**	0.17 (0.08–0.34) **0.00000023**
	TNFSF9 HR (95%CI) *p*-value	0.68 (0.56–0.81) **0.0001**	0.69 (0.57–0.84) **0.00038**	0.47 (0.31–0.71) **0.00075**	0.58 (0.45–0.75) **0.000052**	0.57 (0.39–0.83) **0.017**	1.53 (0.99–2.35) 0.12	2.52 (1.05–6.03) 0.056	0.55 (0.33–0.91) **0.036**
	ULBP1 HR (95%CI) *p*-value	0.68 (0.57–0.81) **0.000088**	0.49 (0.38–0.63) **0.00000015**	0.45 (0.29–0.7) **0.00076**	0.72 (0.56–0.93) **0.016**	0.63 (0.44–0.9) **0.035**	1.52 (0.97–2.38) 0.13	2.08 (1.06–4.09) 0.056	1.48 (0.9–2.43) 0.13
	ULBP2 HR (95%CI) *p*-value	1.33 (0.98–1.8) 0.089	0.82 (0.61–1.12) 0.22	0.68 (0.42–1.12) 0.14	1.77 (1.27–2.46) **0.0011**	1.78 (1.07–2.97) 0.064	0.53 (0.21–1.39) 0.20	1.5 (0.68–3.29) 0.31	3.23 (1.69–6.16) **0.00054**
	ULBP3 HR (95%CI) *p*-value	0.78 (0.61–1) 0.075	1.26 (0.93–1.72) 0.14	0.68 (0.42–1.08) 0.12	0.77 (0.56–1.07) 0.12	1.34 (0.77–2.32) 0.33	3.16 (1.53–6.52) **0.014**	1.7 (0.76–3.8) 0.23	1.72 (0.91–3.26) 0.11
	ULBP4 HR (95%CI) *p*-value	1.15 (0.88–1.5) 0.3	0.66 (0.48–0.93) **0.017**	0.73 (0.46–1.15) 0.18	0.61 (0.43–0.86) **0.0072**	0.63 (0.38–1.05) 0.13	0.53 (0.25–1.12) 0.13	2.86 (1.29–6.31) 0.024	0.69 (0.36–1.31) 0.25
	VIM HR (95%CI) *p*-value	0.79 (0.67–0.94) **0.015**	0.8 (0.65–0.98) **0.032**	0.7 (0.47–1.03) 0.087	1.25 (0.97–1.61) 0.089	0.58 (0.4–0.84) **0.017**	1.22 (0.81–1.83) 0.36	0.36 (0.19–0.69) **0.0074**	0.59 (0.36–0.98) 0.058
NK-activating and inhibitory ligands	HLA-C HR (95%CI) *p*-value	0.7 (0.59–0.84) **0.00032**	0.6 (0.49–0.72) **0.00000083**	0.49 (0.33–0.73) **0.00075**	0.41 (0.31–0.52) **3.14E–12**	0.7 (0.49–0.99) 0.1	0.73 (0.5–1.07) 0.15	0.53 (0.28–1.02) 0.08	0.26 (0.16–0.43) **0.00000023**
	HLA-E HR (95%CI) *p*-value	0.86 (0.72–1.03) 0.11	0.7 (0.56–0.89) **0.0045**	0.54 (0.37–0.79) **0.0032**	0.57 (0.44–0.73) **0.000029**	0.79 (0.55–1.13) 0.24	0.64 (0.44–0.92) 0.077	0.24 (0.12–0.46) **0.000089**	0.48 (0.29–0.78) **0.0059**
	NECL5 HR (95%CI) *p*-value	0.58 (0.47–0.71) **0.0000013**	0.74 (0.6–0.9) **0.0043**	0.45 (0.29–0.69) **0.00071**	0.7 (0.54–0.9) **0.0086**	0.85 (0.6–1.21) 0.38	0.57 (0.34–0.97) 0.11	1.71 (0.86–3.41) 0.15	0.61 (0.35–1.09) 0.11
	NECTIN2 HR (95%CI) *p*-value	0.71 (0.6–0.84) **0.00032**	0.6 (0.49–0.72) **0.00000075**	0.6 (0.41–0.89) **0.013**	0.77 (0.58–1.02) 0.086	1.32 (0.92–1.92) 0.18	0.72 (0.5–1.04) 0.13	0.65 (0.33–1.26) 0.23	1.48 (0.9–2.44) 0.13
NK-inhibitory ligands	CEACAM1 HR (95%CI) *p*-value	0.78 (0.65-0.93) **0.012**	0.7 (0.58-0.86) **0.00085**	0.79 (0.51-1.2) 0.26	0.73 (0.57-0.94) **0.022**	1.52 (1.06-2.16) 0.061	0.69 (0.47-1.01) 0.12	1.5 (0.75-2.98) 0.27	0.6 (0.36-0.97) 0.058
	CLEC2D HR (95%CI) *p*-value	0.46 (0.35- 0.58) **0.0000000083**	0.47 (0.34-0.64) **0.0000042**	0.62 (0.39-1.01) 0.069	0.33 (0.23-0.46) **1.76E-11**	0.6 (0.35-1.01) 0.10	0.61 (0.31-1.21) 0.17	0.62 (0.28-1.4) 0.27	0.48 (0.25-0.93) **0.046**
	COL3A1 HR (95%CI) *p*-value	1.24 (1.04-1.48) **0.022**	1.65 (1.29-2.12) **0.00015**	1.82 (1.17-2.82) **0.01**	1.59 (1.23-2.05) **0.00069**	0.61 (0.42-0.89) **0.031**	1.74 (1.05-2.86) 0.099	1.42 (0.74-2.74) 0.29	1.71 (1.02-2.86) 0.058
	HLA-A HR (95%CI) *p*-value	0.84 (0.69-1.02) 0.091	0.62 (0.51-0.75) **0.000004**	0.45 (0.3-0.68) **0.00058**	0.41 (0.32-0.54) **3.52E-11**	1.2 (0.81-1.78) 0.38	0.64 (0.44-0.93) 0.08	0.38 (0.16-0.92) 0.053	0.29 (0.18-0.48) **0.0000014**
	HLA-B HR (95%CI) *p*-value	0.8 (0.68-0.95) **0.022**	0.61 (0.5-0.74) **0.0000016**	0.45 (0.31-0.67) **0.00038**	0.41 (0.32-0.53) **2.06E-11**	1.18 (0.8-1.73) 0.41	0.73 (0.5-1.06) 0.14	0.52 (0.27-0.99) 0.068	0.27 (0.16-0.44) **0.00000023**
	PDL1 HR (95%CI) *p*-value	0.57 (0.44-0.74) **0.000089**	0.45 (0.32-0.63) **0.0000066**	0.41 (0.26-0.65) **0.00057**	0.27 (0.19-0.37) **< 3.2E-15**	0.5 (0.3-0.85) **0.031**	0.55 (0.28-1.08) 0.13	0.37 (0.17-0.83) **0.041**	0.23 (0.12-0.43) **0.000003**
	PDL2 HR (95%CI) *p*-value	0.8 (0.67-0.95) **0.022**	0.63 (0.52-0.77) **0.000014**	0.57 (0.38-0.86) **0.01**	0.57 (0.42-0.78) **0.00069**	0.8 (0.56-1.14) 0.24	0.76 (0.53-1.1) 0.17	0.38 (0.2-0.72) **0.0092**	0.55 (0.31-0.98) 0.058

Among the NK-activating ligands that were associated with longer RFS in all BC patients (Figure 1), the prognostic values of only CD27, MICB, SLAMF6, TNFSF9, and ULBP1 were found to be independent of the BC subtype as their high expression was associated with longer RFS in all BC subtypes (Table 2). Other ligands were associated with longer RFS in all subtypes but one; luminal A subtype for B7-1, B7-2, CD48, CD72, and SLAMF7 or basal-like subtype for KMT2E (Table 2). However, the prognostic value of the other NK-activating ligands was subtype specific. Particularly, ULBP4 associated with longer RFS specifically in luminal B and basal-like subtypes, CD70 associated with longer RFS specifically in luminal B and HER2-positive subtypes while the favorable prognostic significance of VIM was specifically dependent on luminal A and B subtypes (Table 2). On the other hand, AICL and ULBP2 were associated with shorter RFS specifically in luminal A/B and basal-like subtypes, respectively (Table 2). Contrarily, NECL2, MICA, and BAT3 had opposing prognostic significance depending on the BC subtype (Table 2).

Among the NK-activating ligands that were associated with longer OS in all BC patients, the prognostic values of only CD48 and CD27 were found to be independent of the BC subtype as their expression was associated with longer OS in all BC subtypes (Table 2). Furthermore, SLAMF7 and VIM were associated with longer OS in HER2-positive/basal-like subtypes and luminal A/HER2-positive subtypes, respectively (Table 2). Contrarily, ULBP2 was associated with worse prognosis specifically in basal-like subtype (Table 2).

**Figure 3:**
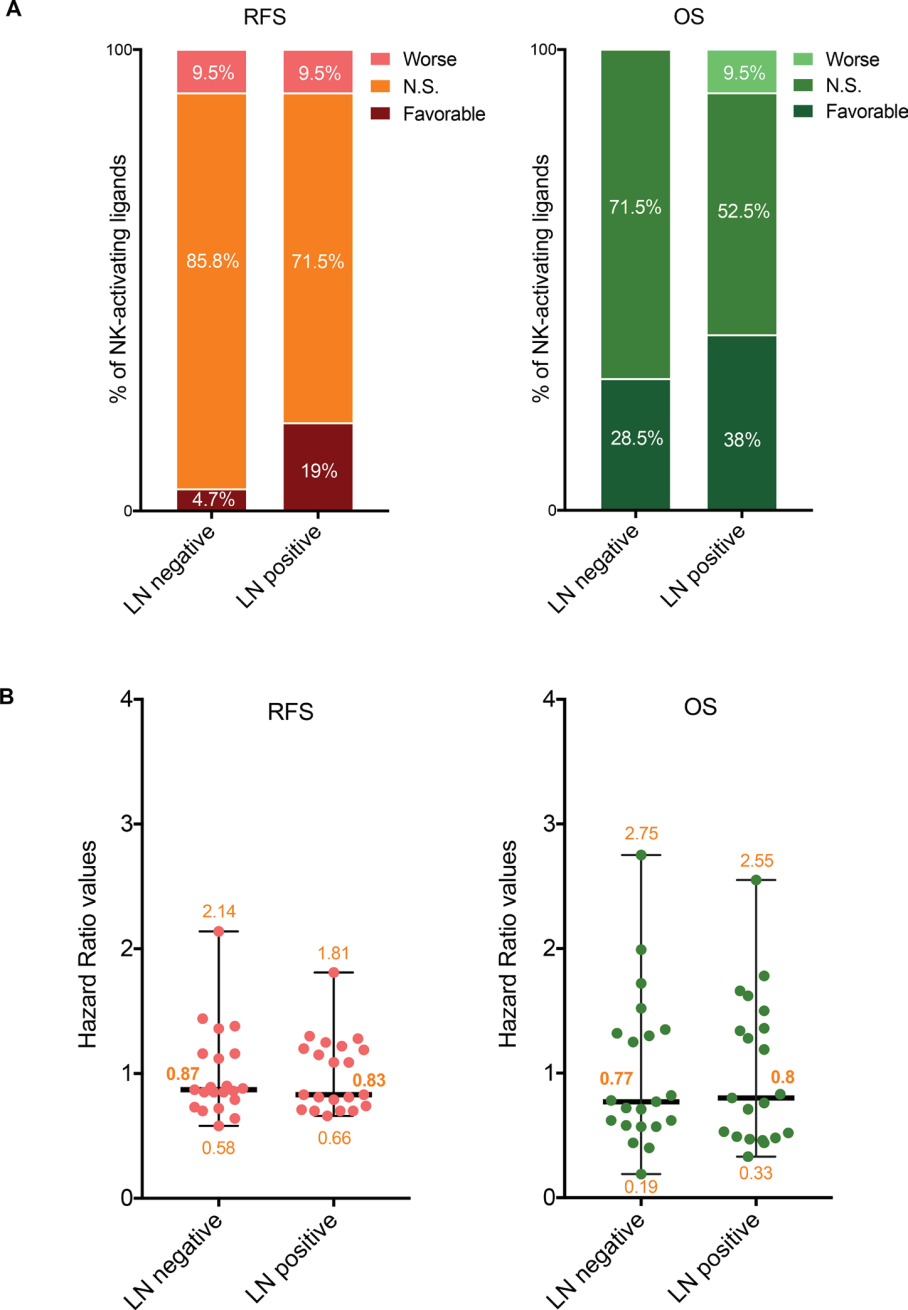
Prognostic values of the mRNA expression of NK-activating ligands depending on the lymph node status (**A**) Percentages of the NK-activating ligands whose high mRNA expression significantly associated (Favorable and Worse) or not (N.S.) with RFS (Left graph) or OS (Right graph) in the different lymph node (LN) statuses. “Favorable“ indicates the association of high mRNA expression with longer RFS/OS, representing good prognosis. “Worse” indicates the association of the high mRNA expression with shorter RFS/OS, representing bad prognosis. “N.S.” indicates no significant association with prognosis. (**B**) Individual value plots visualizing the distribution of the hazard ratio (HR) values of the NK-activating ligands in LN negative and LN positive BC patients. The bars represent the median, lower and higher HR values for all ligands in each subtype.

Taking into account all 21 NK-activating ligands as a whole (Figure 2), we observed that 43%, 66.5%, 66.5% and 52.4% of these ligands correlated with longer RFS in BC patients with tumors of luminal A, luminal B, HER2-positive and basal-like subtypes, respectively (Figure 2A, left graph). When analyzing the percentage of NK-activating ligands whose high expression was associated with longer OS, it dropped to 33.3%, 9.6%, 33.3% and 47.7%, respectively (Figure 3A, right graph). The degree of the loss of the favorable prognostic influence of NK-activating ligands, on OS relative to RFS, varied depending on the BC subtype: slight drop for basal-like subtype (9%), moderate drop for luminal A and HER2-positive subtypes (22.5% and 50% respectively) and dramatic drop for luminal B subtype (85.5% less NK-activating ligands associated with longer OS than with longer RFS) (Figure 2A). Interestingly, when comparing the effect of high mRNA expression of the NK-activating ligands on OS, the HR values appeared to be lower in the basal-like and HER2-positive subtypes in comparison to those of luminal A and B subtypes. In fact, the median HR values were 0.55, 0.57, 0.74, and 0.73 for basal-like, HER2-positive, luminal A and luminal B, respectively (Figure 2B). In other word, patients with HER2-positive or basal-like tumors expressing high mRNA levels of NK-activating ligands were 42–45% less likely to die than patients with low expression levels of these ligands, whereas patients with luminal A/B tumors were 26–27% less likely to die. Thus, high mRNA expression of NK-activating ligands, as a whole, is associated with 16–18% more chance of survival in patients with HER2-positive or basal-like BC than in patients with luminal A or luminal B BC.

On the other hand, except for COL3A1 that correlated with worse RFS in all BC subtypes, all the ligands that bind NK-inhibitory receptors were associated with better RFS mainly in a subtype-independent manner (Table 2). However, the association between these ligands and OS is more variable and subtype-dependent (Table 2).

Taken together, these results indicate that NK receptor ligands, whether activating or inhibitory, are mainly associated with favorable RFS. When taken individually, the prognostic value of some NK receptor ligands is independent of BC subtypes while for others it is more subtype-specific. Interestingly, the favorable prognostic influence of NK-activating ligands’ upregulation, as a whole, is higher in HER2-positive and basal-like BC subtypes.

### Association between the prognostic role of the mRNA expression of NK receptor ligands and the lymph node status of BC patients

The most significant prognostic indicator for patients with early-stage BC is the presence (lymph node positive) or absence (lymph node negative) of axillary lymph node involvement [134]. Therefore, we next checked whether the prognostic influence of NK receptor ligands might be dependent on the lymph node status in BC patients (Table 3 and Figure 3).

**Table 3:** Association between the prognostic role of mRNA expression of NK receptor ligands and lymph-node status

		RFS		OS	
	Ligand	Lymph node negative	Lymph node positive	Lymph node negative	Lymph node positive
**NK-activating ligands**	AICL HR (95%CI) *p*-value	0.89 (0.73–1.08) 0.24	0.74 (0.60–0.91) **0.028**	1.52 (1.05–2.20) 0.069	0.44 (0.30–0.66) **0.0011**
	B7-1 HR (95%CI) *p*-value	1.36 (0.89–2.07) 0.18	0.71 (0.52–0.96) 0.064	0.40 (0.15–1.04) 0.12	0.48 (0.28–0.84) **0.021**
	B7-2 HR (95%CI) *p*-value	1.16 (0.96–1.40) 0.18	1.19 (0.97–1.44) 0.12	0.78 (0.54–1.13) 0.26	0.83 (0.55–1.24) 0.39
	BAT3 HR (95%CI) *p*-value	1.44 (1.21–1.70) **0.00099**	0.81 (0.67–1.00) 0.081	1.32 (0.87–2.02) 0.26	1.50 (1.02–2.21) 0.073
	CD27 HR (95%CI) *p*-value	0.73 (0.62–0.87) **0.0018**	0.7 (0.57–0.85) **0.0024**	0.62 (0.42–0.9) **0.045**	0.46 (0.31–0.68) **0.0011**
	CD48 HR (95%CI) *p*-value	0.87 (0.74–1.03) 0.18	0.79 (0.65–0.96) 0.058	0.71 (0.49–1.04) 0.16	0.47 (0.32–0.70) **0.0011**
	CD58 HR (95%CI) *p*-value	0.79 (0.64-0.96) 0.057	1.2 (1-1.44) 0.081	0.58 (0.39-0.86) **0.044**	1.36 (0.9-2.07) 0.18
	CD70 HR (95%CI) *p*-value	1.16 (0.96–1.40) 0.18	1.30 (1.07–1.59) **0.032**	1.25 (0.86–1.81) 0.27	1.66 (1.10–2.49) **0.034**
	CD72 HR (95%CI) *p*-value	0.86 (0.71–1.04) 0.18	1.09 (0.87–1.37) 0.47	0.82 (0.57–1.19) 0.30	0.52 (0.33–0.81) **0.012**
	KMT2E HR (95%CI) *p*-value	0.7 (0.44–1.1) 0.18	1.15 (0.89–1.48) 0.30	0.19 (0.06–0.59) **0.043**	1.28 (0.76–2.16) 0.39
	MICA HR (95%CI) *p*-value	1.12 (0.93–1.34) 0.24	0.83 (0.67–1.03) 0.12	1.35 (0.91–2.02) 0.24	1.19 (0.81–1.76) 0.39
	MICB HR (95%CI) *p*-value	0.88 (0.74–1.06) 0.22	0.66 (0.54–0.81) **0.00065**	0.77 (0.50–1.20) 0.27	0.49 (0.33–0.72) **0.0018**
	NECL2 HR (95%CI) *p*-value	0.85 (0.72–1.01) 0.13	0.81 (0.66–1.00) 0.081	0.57 (0.40–0.83) **0.043**	0.71 (0.46–1.10) 0.16
	SLAMF6 HR (95%CI) *p*-value	0.64 (0.43–0.94) 0.057	0.70 (0.54–0.90) **0.028**	1.72 (0.68–4.37) 0.27	0.80 (0.46–1.39) 0.44
	SLAMF7 HR (95%CI) *p*-value	0.58 (0.35–0.94) 0.057	0.70 (0.51–0.96) 0.066	0.72 (0.28–1.84) 0.49	0.33 (0.17–0.65) **0.0045**
	TNFSF9 HR (95%CI) *p*-value	0.85 (0.72–1.01) 0.12	1.09 (0.90–1.33) 0.39	0.57 (0.39–0.84) **0.043**	0.76 (0.50–1.15) 0.22
	ULBP1 HR (95%CI) *p*-value	0.85 (0.71–1.03) 0.18	1.22 (0.97–1.55) 0.12	0.62 (0.42–0.91) **0.046**	1.34 (0.91–1.98) 0.17
	ULBP2 HR (95%CI) *p*-value	2.14 (1.45–3.17) **0.001**	1.81 (1.40–2.35) **0.00015**	2.75 (1.10–6.89) 0.069	1.78 (1.05–3.03) 0.062
	ULBP3 HR (95%CI) *p*-value	1.38 (0.89–2.13) 0.18	1.25 (0.95–1.63) 0.12	1.99 (0.66–6.00) 0.27	2.55 (1.49–4.34) **0.0023**
	ULBP4 HR (95%CI) *p*-value	0.72 (0.47–1.10) 0.18	1.28 (0.96–1.72) 0.12	0.44 (0.16–1.23) 0.20	1.62 (0.90–2.93) 0.15
	VIM HR (95%CI) *p*-value	0.9 (0.76–1.06) 0.22	0.83 (0.67–1.03) 0.12	1.3 (0.89–1.88) 0.26	0.53 (0.35–0.79) **0.0064**
**NK-activating and inhibitory ligands**	HLA-C HR (95%CI) *p*-value	0.77 (0.64–0.92) **0.022**	0.82 (0.66 -1.02) 0.10	0.60 (0.41–0.87) **0.044**	0.54 (0.37–0.81) **0.0088**
	HLA-E HR (95%CI) *p*-value	0.72 (0.61–0.86) **0.0012**	0.84 (0.68–1.04) 0.12	0.74 (0.51–1.07) 0.20	0.58 (0.39–0.86) **0.017**
	NECL5 HR (95%CI) *p*-value	0.78 (0.65–0.94) **0.032**	1.26 (0.99–1.59) 0.092	1.27 (0.85–1.88) 0.27	0.62 (0.38–0.99) 0.074
	NECTIN2 HR (95%CI) *p*-value	0.71 (0.60–0.85) **0.001**	0.65 (0.53–0.79) **0.00019**	0.57 (0.37–0.88) **0.045**	0.70 (0.45–1.07) 0.15
**NK-inhibitory ligands**	CEACAM1 HR (95%CI) *p*-value	0.82 (0.69–0.97) 0.057	0.83 (0.68–1.01) 0.10	1.34 (0.89–2.02) 0.26	0.62 (0.41–0.95) 0.062
	CLEC2D HR (95%CI) *p*-value	0.85 (0.57–1.28) 0.44	0.71 (0.55–0.92) **0.032**	0.60 (0.23–1.59) 0.30	1.21 (0.69–2.15) 0.51
	COL3A1 HR (95%CI) *p*-value	0.9 (0.76–1.07) 0.24	0.78 (0.64–0.95) **0.044**	0.79 (0.54–1.17) 0.27	0.65 (0.44–0.96) 0.062
	HLA-A HR (95%CI) *p*-value	0.81 (0.68–0.97) 0.057	0.75 (0.61–0.93) **0.032**	0.70 (0.47–1.03) 0.16	0.71 (0.47–1.08) 0.15
	HLA-B HR (95%CI) *p*-value	0.79 (0.65–0.94) **0.034**	0.79 (0.63–0.98) 0.068	0.61 (0.41–0.90) **0.046**	0.66 (0.44–0.99) 0.074
	PDL1 HR (95%CI) *p*-value	0.51 (0.34–0.77) **0.0052**	0.75 (0.58–0.99) 0.081	0.55 (0.22–1.36) 0.26	0.45 (0.26–0.79) **0.013**
	PDL2 HR (95%CI) *p*-value	0.87 (0.73–1.03) 0.18	0.83 (0.65–1.05) 0.13	1.62 (1.10–2.39) **0.048**	0.72 (0.49–1.06) 0.15

Among all 21 NK-activating ligands, only CD27 was associated with longer RFS in both lymph node negative and positive BC patients (Table 3) while three ligands (AICL, MICB and SLAMF6) were associated with longer RFS specifically in lymph node positive BC patients but not in lymph node negative patients (Table 3). However, none of the NK-activating ligands specifically associated with better RFS in only lymph node negative BC patients (Table 3). On the other hand, three ligands: BAT3 (only in lymph node negative BCs), CD70 (only in lymph node positive BCs) and ULBP2 (in both lymph node negative and positive BCs) correlated with shorter RFS (Table 3). The prognostic significance observed for RFS correlated with OS for AICL, CD27, CD70, and MICB ligands but not for SLAMF6, ULBP2, and BAT3 ligands (Table 3).

Regarding the NK-inhibitory ligands, HLA-B and PDL1 on the one hand and CLEC2D, COL3A1 and HLA-A on the other hand were associated with longer RFS specifically in lymph node negative and lymph node positive BCs, respectively (Table 3). Among these, the association with RFS of only HLA-B correlated with OS (Table 3).

Regarding the ligands that can bind both NK-activating and inhibitory receptors, they all significantly associated with longer RFS in lymph node negative BCs but this was also true in lymph node positive BCs for only NECTIN2 (Table 3). The association between the mRNA expression of these ligands and OS was more variable depending on the lymph node status (Table 3).

Overall, although the median HR values for all NK-activating ligands were similar between lymph node positive and lymph node negative BCs for both RFS and OS (Figure 3B), more NK activating ligands were associated with better prognosis in lymph node positive BC patients (4–8/21, 19–38%) than in lymph node negative BC patients (1–6/21, 4.7–28.1%), for RFS-OS, respectively (Figure 3A). However, no tendency could be observed for the ligands that can bind NK-inhibitory receptors.

### Association between the prognostic role of the mRNA expression of NK receptor ligands and the BC pathological grade

**Figure 4:**
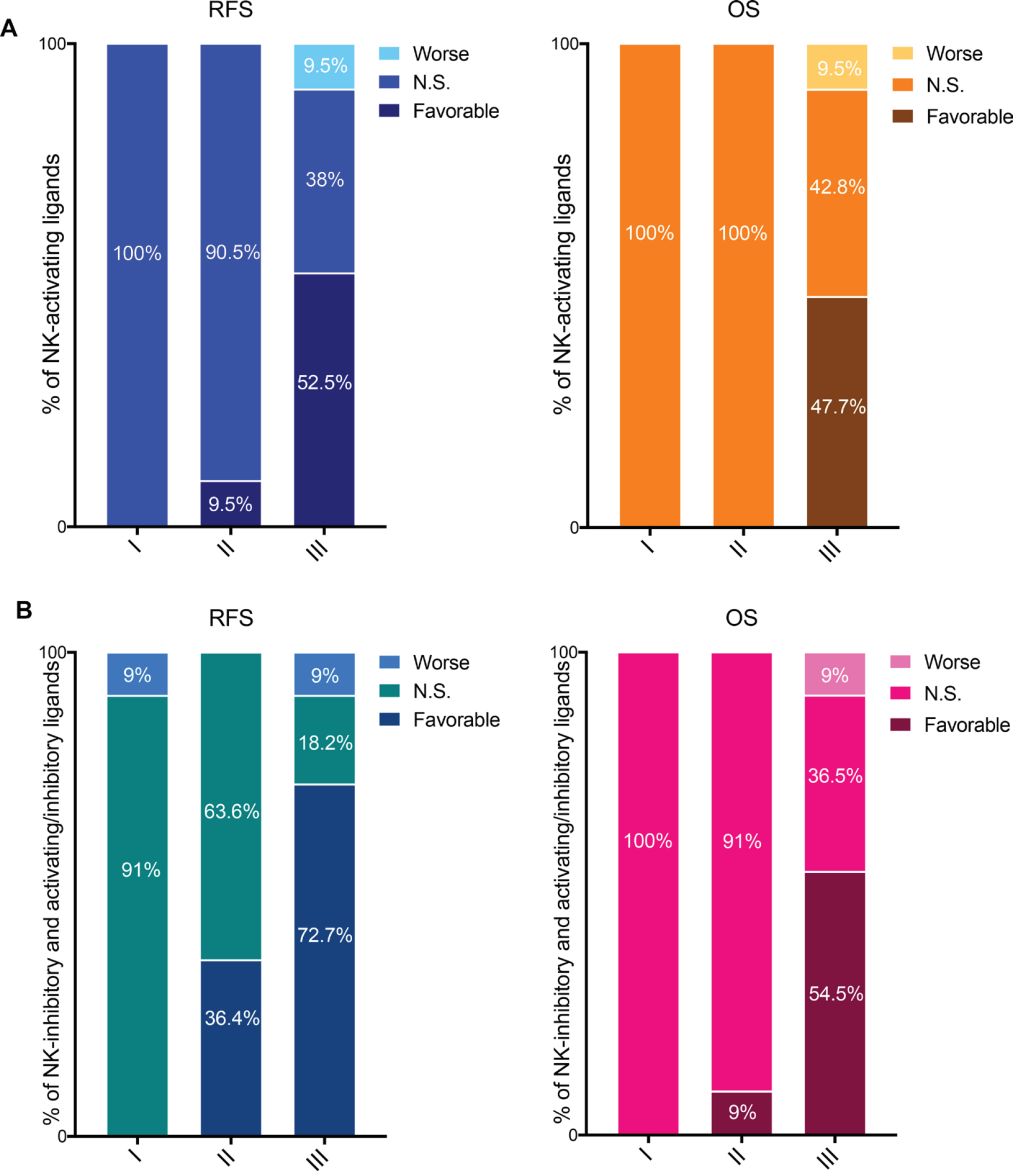
Prognostic values of the mRNA expression of NK-activating ligands depending on the tumor pathological grade (**A**) Percentages of the NK-activating ligands whose high mRNA expression significantly associated (Favorable and Worse) or not (N.S.) with RFS (Left graph) or OS (Right graph) in the different tumor grades. “Favorable“ indicates the association of high mRNA expression with longer RFS/OS, representing good prognosis. “Worse” indicates the association of the high mRNA expression with shorter RFS/OS, representing bad prognosis. “N.S.” indicates no significant association with prognosis. (**B**) Same as in panel “a” but for NK-inhibitory ligands together with the ligands that can bind both NK-activating and inhibitory receptors (i.e. NK-activating/inhibitory ligands).

In addition to the molecular subtype and lymph node status, the tumor pathological grade is another factor that affects treatment choice and cancer patient prognosis. In BC, grades I, II and III are ascending indicators of how quickly a tumor is likely to grow and spread by describing the abnormality of the tumor tissue based on the tubular differentiation, nuclear features and mitotic activity of tumor cells. Therefore, we also checked whether the prognostic influence of NK receptor ligands might be affected by the breast tumor grade (Table 4 and Figure 4).

**Table 4:** Association between the prognostic role of mRNA expression of NK receptor ligands and tumor grade

	Ligand	RFS			OS		
		I	II	III	I	II	III
**NK-activating ligands**	AICL HR (95%CI) *p*-value	0.69 (0.39–1.22) 0.29	0.71 (0.56–0.90) **0.039**	0.77 (0.62–0.96) **0.0303**	1.73 (0.66–4.56) 0.33	0.49 (0.27–0.87) 0.076	0.64 (0.46–0.89) **0.016**
	B7-1 HR (95%CI) *p*-value	1.72 (0.60–4.97) 0.34	0.70 (0.42–1.17) 0.209	0.53 (0.39–0.72) **0.00018**	0.14 (0.01–1.64) 0.17	0.47 (0.15–1.48) 0.22	0.33 (0.18–0.58) **0.00022**
	B7-2 HR (95%CI) *p*-value	0.62 (0.37–1.05) 0.24	0.83 (0.66–1.06) 0.209	0.74 (0.57–0.95) **0.032**	0.76 (0.31–1.90) 0.56	0.72 (0.47–1.10) 0.19	0.68 (0.48–0.95) **0.040**
	BAT3 HR (95%CI) *p*-value	1.38 (0.80–2.37) 0.33	1.40 (1.08–1.82) 0.054	1.23 (0.95–1.58) 0.12	2.48 (0.72–8.55) 0.22	1.62 (1.04–2.52) 0.099	1.38 (0.94–2.04) 0.13
	CD27 HR (95%CI) *p*-value	0.63 (0.37–1.06) 0.24	0.72 (0.56–0.91) **0.039**	0.58 (0.46–0.73) **0.000016**	0.31 (0.11–0.88) 0.15	0.69 (0.45–1.05) 0.14	0.44 (0.32–0.61) **0.0000061**
	CD48 HR (95%CI) *p*-value	0.71 (0.42–1.18) 0.29	0.74 (0.58–0.94) 0.056	0.60 (0.46–0.77) **0.00022**	0.45 (0.18–1.11) 0.17	0.74 (0.48–1.14) 0.22	0.44 (0.31–0.61) **0.0000061**
	CD58 HR (95%CI) *p*-value	0.71 (0.42-1.19) 0.29	0.75 (0.56-0.99) 0.102	0.74 (0.59-0.92) **0.014**	2.29 (0.92-5.7) 0.17	0.59 (0.38-0.92) 0.076	0.74 (0.54-1.03) 0.11
	CD70 HR (95%CI) *p*-value	0.76 (0.44–1.31) 0.34	0.81 (0.60–1.09) 0.209	0.78 (0.60–1.02) 0.088	0.40 (0.11–1.37) 0.22	1.81 (1.17–2.78) 0.076	0.78 (0.55–1.12) 0.21
	CD72 HR (95%CI) *p*-value	1.71 (1.01–2.90) 0.22	0.88 (0.69–1.11) 0.28	0.70 (0.53–0.91) **0.018**	2.21 (0.90–5.43) 0.17	0.74 (0.48–1.14) 0.22	0.52 (0.35–0.77) **0.0028**
	KMT2E HR (95%CI) *p*-value	4.18 (0.55–31.97) 0.27	0.65 (0.38–1.09) 0.18	1.49 (1.09–2.04) **0.0207**	0 (0–inf) 0.17	0.13 (0.02–0.98) 0.076	1.42 (0.78–2.58) 0.27
	MICA HR (95%CI) *p*-value	1.30 (0.76–2.23) 0.35	1.16 (0.92–1.48) 0.23	1.57 (1.26–1.97) **0.00022**	2.09 (0.80–5.43) 0.22	0.67 (0.43–1.05) 0.14	1.97 (1.41–2.75) **0.00022**
	MICB HR (95%CI) *p*-value	0.71 (0.41–1.20) 0.29	0.76 (0.58–0.99) 0.102	0.73 (0.59–0.91) **0.0105**	0.57 (0.23–1.43) 0.32	0.75 (0.49–1.15) 0.22	0.64 (0.46–0.89) **0.017**
	NECL2 HR (95%CI) *p*-value	0.47 (0.28–0.80) 0.065	0.75 (0.59–0.96) 0.074	1.21 (0.97–1.51) 0.107	0.25 (0.07–0.86) 0.15	0.60 (0.39–0.93) 0.078	1.26 (0.90–1.75) 0.209
	SLAMF6 HR (95%CI) *p*-value	0.42 (0.14–1.20) 0.24	0.67 (0.40–1.12) 0.209	0.52 (0.38–0.71) **0.00012**	0 (0–inf) 0.17	5.84 (0.75–45.29) 0.11	0.76 (0.45–1.28) 0.309
	SLAMF7 HR (95%CI) *p*-value	1.84 (0.58–5.86) 0.34	0.68 (0.41–1.13) 0.209	0.51 (0.37–0.70) **0.00012**	0.21 (0.02–2.29) 0.22	0.47 (0.15–1.50) 0.22	0.31 (0.18–0.55) **0.00012**
	TNFSF9 HR (95%CI) *p*-value	0.73 (0.43–1.22) 0.32	0.83 (0.64–1.07) 0.209	0.75 (0.60–0.94) **0.0207**	0.57 (0.21–1.52) 0.33	1.56 (0.99–2.46) 0.11	0.67 (0.47–0.95) **0.0404**
	ULBP1 HR (95%CI) *p*-value	0.65 (0.34–1.23) 0.29	0.84 (0.66–1.07) 0.209	0.84 (0.67–1.05) 0.12	0.47 (0.17–1.34) 0.22	1.36 (0.88–2.11) 0.22	0.80 (0.54–1.19) 0.28
	ULBP2 HR (95%CI) *p*-value	0.49 (0.17–1.47) 0.29	1.80 (1.06–3.06) 0.081	1.46 (1.04–2.05) **0.037**	7.04 (0.61–81.25) 0.17	0.24 (0.05–1.11) 0.11	2.05 (1.20–3.48) **0.016**
	ULBP3 HR (95%CI) *p*-value	0.69 (0.23–2.07) 0.51	1.22 (0.73–2.04) 0.44	1.28 (0.93–1.75) 0.13	0.24 (0.01–3.78) 0.33	3.65 (1.17–11.39) 0.076	2.07 (1.05–4.08) 0.052
	ULBP4 HR (95%CI) *p*-value	0.39 (0.12–1.23) 0.24	1.43 (0.86–2.37) 0.209	0.77 (0.55–1.09) 0.14	0 (0–inf) 0.15	0.54 (0.16–1.81) 0.33	1.43 (0.81–2.52) 0.25
	VIM HR (95%CI) *p*-value	0.76 (0.44–1.31) 0.34	1.2 (0.91–1.58) 0.23	0.83 (0.65–1.05) 0.12	2.18 (0.89–5.35) 0.17	0.66 (0.43–1.03) 0.12	0.64 (0.46–0.91) **0.022**
**NK-activating and inhibitory ligands**	HLA-C HR (95%CI) *p*-value	1.83 (1.08–3.10) 0.18	0.76 (0.59–0.97) 0.081	0.54 (0.44–0.67) **0.000000768**	0.62 (0.25–1.59) 0.37	1.25 (0.78–2.01) 0.36	0.42 (0.30–0.58) **0.0000025**
	HLA-E HR (95%CI) *p*-value	0.65 (0.39–1.09) 0.24	0.72 (0.56–0.92) **0.042**	0.67 (0.54–0.84) **0.00088**	0.54 (0.22–1.35) 0.26	0.65 (0.42–1.00) 0.11	0.50 (0.36–0.69) **0.00012**
	NECL5 HR (95%CI) *p*-value	1.33 (0.77–2.30) 0.34	0.68 (0.52–0.88) **0.039**	0.79 (0.63–0.98) **0.041**	0.67 (0.25–1.77) 0.43	1.8 (1.1–2.95) 0.076	0.72 (0.5–1.05) 0.12
	NECTIN2 HR (95%CI) *p*-value	0.62 (0.32–1.19) 0.29	0.64 (0.50–0.81) **0.008**	0.82 (0.65–1.03) 0.11	1.64 (0.52–5.12) 0.43	0.70 (0.46–1.09) 0.18	1.28 (0.92–1.79) 0.17
**NK-inhibitory ligands**	CEACAM1 HR (95%CI) *p*-value	1.79 (1.03–3.13) 0.22	1.23 (0.96–1.57) 0.18	0.77 (0.61–0.97) **0.037**	1.51 (0.60–3.80) 0.42	1.37 (0.87–2.16) 0.22	0.76 (0.53–1.08) 0.17
	CLEC2D HR (95%CI) *p*-value	1.90 (0.53–6.81) 0.34	1.50 (0.87–2.58) 0.209	0.76 (0.54–1.07) 0.12	0 (0–inf) 0.17	0.58 (0.17–1.94) 0.37	1.25 (0.70–2.21) 0.45
	COL3A1 HR (95%CI) *p*-value	0.45 (0.25–0.82) 0.076	0.71 (0.56–0.91) **0.039**	1.55 (1.24–1.93) **0.00023**	0.33 (0.12–0.91) 0.15	0.5 (0.32–0.76) **0.038**	1.66 (1.18–2.34) **0.0085**
	HLA-A HR (95%CI) *p*-value	1.59 (0.93–2.73) 0.24	0.81 (0.64–1.03) 0.18	0.56 (0.45–0.70) **0.0000035**	0.67 (0.26–1.78) 0.43	1.32 (0.83–2.10) 0.26	0.49 (0.35–0.68) **0.000096**
	HLA-B HR (95%CI) *p*-value	2.36 (1.41–3.95) **0.025**	0.77 (0.60–0.98) 0.082	0.58 (0.46–0.72) **0.000011**	2.16 (0.87–5.41) 0.18	0.58 (0.37–0.9) 0.076	0.51 (0.36–0.71) **0.00023**
	PDL1 HR (95%CI) *p*-value	0.40 (0.14–1.15) 0.24	0.75 (0.45–1.25) 0.28	0.46 (0.34–0.63) **0.0000075**	0 (0–inf) 0.15	0.53 (0.17–1.68) 0.29	0.33 (0.18–0.60) **0.00032**
	PDL2 HR (95%CI) *p*-value	1.53 (0.91–2.57) 0.25	1.19 (0.92–1.53) 0.22	0.76 (0.61–0.95) **0.024**	2.40 (0.94–6.09) 0.17	1.75 (1.02–3.02) 0.11	0.67 (0.48–0.93) **0.0301**

The NK-activating ligands B7-1, B7-2, CD48, CD58, CD72, MICB, SLAMF6, SLAMF7 and TNFSF9 were associated with longer RFS specifically in grade III tumors while AICL and CD27 were associated with longer RFS in grade II and grade III tumors. None of the NK-activating ligands was of favorable prognostic value in grade I tumors. The longer RFS that was associated with high mRNA expression of NK-activating ligands strongly correlated with longer OS for AICL (grade III only), B7-1, B7-2, CD27 (grade III only), CD48, CD72, MICB, SLAMF7 and TNFSF9 (Table 4). On the other hand, high mRNA expression of MICA associated with shorter RFS and OS specifically in grade III tumors, whereas ULBP2 was associated with shorter OS specifically in grade III tumors (Table 4).

Overall, 11–10/21 (52.5–47.7%) NK-activating ligands were associate with favorable RFS-OS in grade III BCs, 2–0/21 (9.5–0%) in grade II and 0–0/20 (0–0%) in grade I (Figure 4A).

Among the NK-inhibitory ligands, HLA-B was significantly associated with shorter RFS in grade I tumors but with longer RFS in grade III tumors. On the contrary, COL3A1 was significantly associated with shorter RFS and OS in grade III tumors but with longer RFS and OS in grade II tumors. On the other hand, HLA-A, PDL1, and PDL2 associated with longer RFS and OS specifically in grade III tumors (Table 4).

The NK-activating and inhibitory ligands were significantly associated with longer RFS in grade II and/or grade III tumors (Table 4).

Taken together, as per NK-activating ligands, the favorable prognostic values of most NK-inhibitory and NK-activating and inhibitory ligands were associated with high grade BCs; 0–0%, 36.4–9% and 72.7–54.5% of these ligands associated with longer RFS-OS specifically in grade I, II and III BCs, respectively (Figure 4B).

### Association between the prognostic role of the mRNA expression of NK receptor ligands and the p53 status in BC patients

In BC, mutations in the tumor suppressor gene p53 are present in 18–25% of primary BCs and are associated with more aggressive disease and worse prognosis [135, 136]. In order to test whether the prognostic value of NK receptor ligands might be affected by the p53 status, the association between mRNA expression of these ligands and survival were analyzed in patients with p53 wild-type and p53 mutated BCs (Table 5).

**Table 5:** Association between the prognostic role of mRNA expression of NK receptor ligands and p53 status

		RFS		OS	
	Ligand	Wild-type	Mutated	Wild-type	Mutated
**NK-activating ligands**	AICL HR (95%CI) *p*-value	0.50 (0.32–0.78) **0.042**	0.51 (0.31–0.83) **0.016**	0.42 (0.22–0.81) 0.068	0.44 (0.2–0.94) 0.073
	B7-1 HR (95%CI) *p*-value	0.66 (0.27–1.66) 0.38	0.37 (0.20–0.66) **0.0026**	N/A N/A	0.29 (0.07–1.16) 0.14
	B7-2 HR (95%CI) *p*-value	1.35 (0.85–2.13) 0.26	0.46 (0.28–0.77) **0.0092**	0.65 (0.33–1.30) 0.24	0.37 (0.17–0.79) **0.028**
	BAT3 HR (95%CI) *p*-value	1.27 (0.81–1.98) 0.35	0.63 (0.36–1.11) 0.15	0.60 (0.31–1.14) 0.23	2.06 (0.71–5.96) 0.23
	CD27 HR (95%CI) *p*-value	0.71 (0.45–1.12) 0.23	0.39 (0.24–0.65) **0.0012**	0.67 (0.35–1.28) 0.24	0.29 (0.13–0.61) **0.0081**
	CD48 HR (95%CI) *p*-value	0.55 (0.36–0.84) **0.042**	0.52 (0.31–0.85) **0.019**	0.63 (0.32–1.24) 0.24	0.32 (0.15–0.69) **0.011**
	CD58 HR (95%CI) *p*-value	0.55 (0.36-0.85) **0.042**	0.66 (0.4-1.09) 0.14	0.48 (0.25-0.92) 0.11	0.5 (0.22-1.12) 0.15
	CD70 HR (95%CI) *p*-value	1.41 (0.84–2.38) 0.26	0.76 (0.46–1.25) 0.29	1.65 (0.84–3.24) 0.24	0.51 (0.24–1.10) 0.15
	CD72 HR (95%CI) *p*-value	0.81 (0.52–1.26) 0.37	0.35 (0.18–0.69) **0.0064**	1.53 (0.80–2.92) 0.24	0.29 (0.14–0.63) **0.0081**
	KMT2E HR (95%CI) *p*-value	2.05 (0.87–4.8) 0.22	0.7 (0.38–1.31) 0.28	N/A N/A	1.66 (0.44–6.18) 0.45
	MICA HR (95%CI) *p*-value	0.59 (0.39–0.90) **0.048**	1.33 (0.83–2.15) 0.26	0.55 (0.28–1.08) 0.19	2.76 (1.21–6.32) **0.038**
	MICB HR (95%CI) *p*-value	0.55 (0.35–0.86) **0.042**	0.55 (0.33–0.91) **0.033**	0.41 (0.21–0.82) 0.068	0.52 (0.23–1.17) 0.17
	NECL2 HR (95%CI) *p*-value	0.62 (0.38–1.01) 0.16	1.26 (0.78–2.02) 0.35	0.47 (0.24–0.92) 0.11	0.71 (0.33–1.53) 0.39
	SLAMF6 HR (95%CI) *p*-value	2.01 (0.73–5.52) 0.25	0.36 (0.17–0.74) **0.012**	N/A N/A	2.01 (0.54–7.49) 0.34
	SLAMF7 HR (95%CI) *p*-value	0.54 (0.20–1.47) 0.28	0.22 (0.11–0.45) **0.000088**	N/A N/A	0.19 (0.05–0.72) **0.025**
	TNFSF9 HR (95%CI) *p*-value	1.74 (1.01–2.99) 0.15	1.20 (0.71–2.03) 0.5	1.82 (0.76–4.36) 0.24	0.64 (0.26–1.54) 0.35
	ULBP1 HR (95%CI) *p*-value	0.64 (0.40–1.03) 0.19	1.64 (0.95–2.84) 0.12	0.62 (0.32–1.19) 0.24	1.96 (0.91–4.25) 0.15
	ULBP2 HR (95%CI) *p*-value	1.99 (0.81–4.92) 0.23	2.29 (1.27–4.12) **0.013**	N/A N/A	17.36 (2.17–139.12) **0.0073**
	ULBP3 HR (95%CI) *p*-value	5.76 (1.34–24.78) **0.042**	0.64 (0.33–1.22) 0.21	N/A N/A	1.66 (0.43–6.38) 0.45
	ULBP4 HR (95%CI) *p*-value	3.19 (0.75–13.66) 0.22	0.61 (0.31–1.20) 0.2	N/A N/A	0.25 (0.03–2.04) 0.23
	VIM HR (95%CI) *p*-value	0.71 (0.46–1.10) 0.23	1.5 (0.81–2.80) 0.24	0.52 (0.27–1.00) 0.13	0.64 (0.29–1.41) 0.34
**NK-activating and inhibitory ligands**	HLA-C HR (95%CI) *p*-value	0.78 (0.50–1.23) 0.35	0.47 (0.29–0.76) **0.0064**	0.64 (0.33–1.22) 0.24	0.33 (0.15–0.73) **0.017**
	HLA-E HR (95%CI) *p*-value	0.56 (0.36–0.85) **0.042**	0.40 (0.24–0.67) **0.0019**	0.49 (0.25–0.96) 0.13	0.32 (0.15–0.68) **0.011**
	NECL5 HR (95%CI) *p*-value	0.80 (0.49–1.28) 0.37	1.75 (0.92–3.33) 0.13	0.49 (0.24–1.01) 0.13	1.58 (0.71–3.51) 0.34
	NECTIN2 HR (95%CI) *p*-value	0.73 (0.48–1.11) 0.23	0.72 (0.42–1.23) 0.26	1.69 (0.86–3.34) 0.24	1.58 (0.68–3.68) 0.34
**NK-inhibitory ligands**	CEACAM1 HR (95%CI) *p*-value	1.37 (0.88–2.14) 0.25	0.53 (0.31–0.90) **0.033**	1.57 (0.76–3.24) 0.24	0.52 (0.24–1.13) 0.15
	CLEC2D HR (95%CI) *p*-value	2.31 (0.90–5.91) 0.19	0.43 (0.19–0.96) 0.0604	N/A N/A	4.87 (1.01–23.45) 0.073
	COL3A1 HR (95%CI) *p*-value	0.57 (0.37–0.88) **0.045**	1.69 (0.9–3.15) 0.14	0.32 (0.16–0.62) **0.0078**	0.53 (0.21–1.33) 0.23
	HLA-A HR (95%CI) *p*-value	1.21 (0.79–1.88) 0.38	0.56 (0.34–0.90) **0.033**	0.74 (0.37–1.50) 0.4	0.52 (0.24–1.13) 0.15
	HLA-B HR (95%CI) *p*-value	0.75 (0.49–1.15) 0.26	0.40 (0.25–0.65) **0.00106**	0.57 (0.30–1.12) 0.22	0.32 (0.15–0.68) **0.011**
	PDL1 HR (95%CI) *p*-value	0.45 (0.15–1.33) 0.23	0.20 (0.11–0.37) **0.00000064**	N/A N/A	0.22 (0.06–0.82) **0.0407**
	PDL2 HR (95%CI) *p*-value	0.79 (0.49–1.28) 0.37	0.56 (0.35–0.91) **0.033**	0.73 (0.38–1.40) 0.36	0.61 (0.23–1.64) 0.35

The prognostic values of individual NK-activating ligands differentially associated with the p53 status (Table 5). For example, AICL, CD48, and MICB were associated with longer RFS in both p53 wild-type and p53 mutated BCs (Table 5) which correlated with longer OS for only CD48 (p53 mutated). However, B7-1, B7-2, CD27, CD72, SLAMF6 and SLAMF7 were associated with longer RFS specifically in p53 mutated BCs, which correlated with OS for all except for B7–1 and SLAMF6. In contrast, high mRNA expression of MICA correlated with longer RFS specifically in p53 wild-type BCs without any significant association with OS. As for the NK-activating ligands that correlated with worse prognosis, ULBP3 on the one hand and ULBP2 on the other hand were associated with shorter RFS specifically in p53 wild-type or p53 mutated BCs, respectively (Table 5). Regarding the ligands that can bind NK-inhibitory receptors, they were mostly associated with longer RFS in p53 mutated BCs (Table 5).

## DISCUSSION

Despite a significant piece of experimental and clinical evidence supporting the role of NK cells in BC control, BC still develops and progresses to form large tumors and metastases [22, 25–33]. Several mechanisms of cancer escape from NK immunity were proposed [137]. Among these, BC cells modulate their immunogenicity mainly by altering the expression of ligands for NK cell activating and inhibitory receptors; thereby stimulating a state of immunological tolerance by rendering themselves invisible to NK cells. This mechanism of cancer cell escape from NK immunity is frequently observed in solid tumors including BC [137], which suggests that NK receptor ligands’ expression may have prognostic significance in BC patients and may help identify candidates for NK-based immunotherapies. Therefore, in the present study, we firstly performed systematic literature screening to identify and select all NK-regulatory ligands for NK receptors known to date. In total, we identified 39 ligands for NK activating and inhibitory receptors. Then, we utilized the KM plotter platform to investigate whether the expression of these ligands may influence RFS and OS, and predict prognosis in BC patients and whether these effects may differ by molecular subtypes and other clinicopathological features.

Among the 21 analyzed NK-activating ligands, the high expression of 16 (80%) ligands significantly correlated with better RFS in all BCs, suggesting a protective role of these ligands against cancer progression. However, while the longer RFS correlated with longer OS for about half of these NK-activating ligands, the results showed no significant difference in the OS between the two groups of patients with different expression levels of B7-1, B7-2, CD70, MICB, TNFSF9, ULBP1, and ULBP4 or showed worse OS for high expression of MICA and ULBP3 ligands. This absence of correlation between better RFS and OS can be the consequence of the limited efficacy of the second line therapy in the group of patients with high expression of these ligands. This limited treatment efficacy can also be the result of a first line therapy-induced selective advantage of recurrent tumors that are resistant to the subsequent treatments, thereby accelerating cancer progression and patient death. Therefore, patient management should be optimized after relapse or even before to prevent later recurrence of more aggressive tumor. In this regard, NK-based therapy such as the adoptive transfer of NK cells expressing (endogenously or by genetic engineering) the activating receptors for these ligands might be a potential strategy to improve OS of these patients. However, if the treatment is considered after relapse, the maintenance of the expression of the NK receptor ligands in the secondary tumor should be tested. Furthermore, as mRNA expression does not necessarily correlate with protein expression in all cases, the expression of a considered ligand should also be tested at the protein level to predict a potential response to an NK-based treatment.

On the other hand, other NK-activating ligands (i.e. BAT3, CD58, and ULBP2) were unexpectedly associated with worse prognosis suggesting that these ligands are markers of more aggressive tumors. Although the high expression of these ligands is expected to enhance tumor cell elimination by NK cells, these lymphocytes might be either absent in these tumors or unresponsive to these ligands. The possible unresponsiveness of NK cells to these ligands expressed by cancer cells can be the consequence of tumor-induced deregulation of the expression of their cognate receptors on the surface of the NK cells. In fact, BC cells can release immunosuppressive molecules such as transforming growth factor-β1 (TGF- β1) and soluble MICAs that can downregulate the activating receptors and upregulate the inhibitory receptors on NK cells, as a mechanism of tumor escape from immune surveillance [137]. Another hypothesis might be that in addition to their role as ligands for NK-activating receptors, these genes might also have another NK-independent pro-oncogenic function, which confers increased aggressiveness to the tumor cells. Accordingly, BAT3 has been shown to play a role in the induction of the cell cycle progression by regulating p21 protein [138] and protection from apoptosis by inducing the anti-apoptotic YWK-II/APLP2 protein stability [139]. Furthermore, CD58 can promote the self-renewal of tumor-initiating cells by upregulating the Wnt/β-catenin pathway [140]. However, to our knowledge, no NK-independent oncogenic function of ULBP2 has been identified to date. Whatever the mechanism involved, the association of these NK-activating ligands with shorter time to relapse and survival suggests that BC patients expressing high levels of BAT3, CD58, or ULBP2 may be favorable candidates for NK-cell based therapy. A possible therapy would be the adoptive transfer of NK cells expressing receptors for these ligands (i.e. NKp30, CD2, or NKG2D) to induce the elimination of residual tumor cells, prevent relapse and improve patient survival.

Since the activity of NK cells is negatively regulated by the engagement of their inhibitory receptors, high expression of the NK-inhibitory ligands (CEACAM1, CLEC2D, HLA-A, HLA-B, PDL1, and PDL2) was expected to be associated with worse prognosis. However, except for COL3A1, the NK-inhibitory ligands were associated with favorable prognosis, suggesting that these ligands might have a cancer-protective role in addition to their function as NK-inhibitory ligands. Indeed, the expression of MHC class I molecules (HLA-A, HLA-B, and HLA-C) on cancer cells allows their detection and destruction by T cell lymphocytes [141]. Accordingly, downregulation or loss of these molecules in BC and other cancers increases metastasis to the lymph nodes and other organs [142]. Furthermore, CEACAM1 is an adhesion molecule that is regarded as a tumor suppressor and was found to regulate tumor growth and apoptosis in many types of cancer including BC [143, 144]. On the other hand, although PDL1 expression by tumor cells is believed to mediate inhibition of local immune response by down-modulating tumor-infiltrating lymphocyte (TIL), including NK and T cell, function, survival, and expansion [145], our study defines high PDL1 expression as a positive prognostic biomarker in BC, in agreement with other studies [146, 147]. This survival result might be due to the presence of a strong antitumor immune response leading to PDL1 expression. In fact, it has been shown that TILs can release cytokines including interferon-γ that upregulate PDL1 expression on tumor cells [145, 146, 148–150]; thus indicating a strong anti-tumor immune response.

Importantly, our finding that high expression of most ligands for NK-inhibitory receptors is associated with favorable prognosis and accordingly low expression of these ligands is associated with worse prognosis is of high therapeutic interest. In fact, this finding suggests that in patients with worse prognosis, the low expression of NK-inhibitory ligands would reduce the inhibitory signals for NK cell activation and enhance their cytotoxic potential towards tumor cells; thereby increasing the chances of response to NK-based therapy. Accordingly, we suggest that BC patients with tumors expressing high levels of the NK-activating ligands that would mark worse prognosis or limited OS (such as BAT3, CD58, ULBP2, MICA, ULBP3, B7-1, B7-2, CD70, MICB, TNFSF9, ULBP1 and ULBP4), would probably have tumors with low expression of NK-inhibitory ligands (which should be tested and confirmed in the tumor), making them ideal candidates for a successful NK-based immunotherapy.

Analysis of the association between the prognostic role of the mRNA expression of NK receptor ligands and the different BC subtypes showed that the prognostic influence of CD27, CD48, MICB, SLAMF6, TNFSF9, ULBP1, HLA-C, NECL5, COL3A1, HLA-B, PDL1, and PDL2 is independent of the BC molecular subtype whereas the effect of the other NK receptor ligands on patient relapse and survival may vary between the different BC subtypes. Interestingly, the favorable prognostic influence of NK-activating ligands, as a whole, is higher in basal-like and HER2 types in comparison to luminal A/B. This is probably due to the different types of conventional therapeutics used in the different BC subtypes. In fact, chemotherapy (by further enhancing the expression of NK-activating ligands or reducing the expression of NK-inhibitory ligands on tumor cells) [151] and HER2-targeted therapy (by Trastuzumab-induced NK cell-based antibody-dependent cell-mediated cytotoxicity, i.e. ADCC) [152] were shown to increase cancer cell sensitivity to NK-mediated cytotoxicity which could act synergistically with the basal high expression of NK-activating ligands in basal-like and/or HER2-positive BCs. In contrast, tamoxifen, which is widely used in endocrine therapy for ER-positive (luminal A and B) BCs, was shown to inhibit NK-mediated BC cell death by inducing the expression of the granzyme B inhibitor (serpentinB9/proteinase inhibitor 9), making these cells less responsive to NK despite the high expression of NK-activating ligands on their surface [153]. Furthermore, we could observe that the favorable prognostic influence of NK-activating ligands was significantly higher in lymph node positive and grade III BCs than in lymph node negative and lower grades BCs. This could be the consequence of a higher NK cell infiltration of lymph node positive and grade III BCs than in lymph node negative and lower grades BCs [154, 155]. Thus, therapeutic approaches that can harness the cytotoxic potential of these lymphocytes might improve tumor management and survival in BC patients with lymph node involvement and/or grade III tumors that are; therefore, initially classed as having a poor prognosis.

In BC, NK cell-based immunotherapy can have four main approaches [24]: 1) direct administration of these immune cells genetically modified and/or stimulated *ex vivo*. 2) administration of drugs, mainly cytokines, to stimulate NK cells in patients themselves. 3) targeting therapies with monoclonal antibodies (such as trastuzumab for HER2-positive breast cancer that triggers NK cell-mediated ADCC. 4) use of immunomodulatory drugs such as TGF- β1 or TGF- β1 receptors inhibitors or blocking antibodies for NK-inhibitory receptors.

An increasing number of research studies trying to harness NK cell function against cancer cells were recently performed [24, 31, 32, 156, 157]. However, to date, in contrast to other types of cancer such as leukemia, neuroblastoma and glioblastoma, clinical trials using NK cell-based immunotherapy in BC failed to improve clinical outcomes [24]. Therefore, in order to develop effective anti-BC immunotherapy approaches and benefit from the high anti-tumoral potential of NK cells and their safety towards healthy tissues, it’s crucial to determine and consider predictive biomarkers for NK-therapy responsiveness in BC patients.

In conclusion, in BC, all NK receptor ligands were found to be of valuable potential prognostic biomarkers, that can or cannot be affected by the different BC subtypes or clinicopathological features depending on the individual ligand considered. The favorable prognostic influence of NK-activating ligands’ upregulation, as a whole, was mainly significantly associated with HER2-positive and basal-like subtypes, lymph node positive phenotype and high-grade tumors. Furthermore, we identified two groups of BC patients with specific expressions of NK-activating ligands as potential candidates for NK-based therapy such as adoptive transfer of NK cells expressing receptors for these ligands. Those include patients with tumors whose NK-activating ligand is associated with higher RFS but no effect or worse OS as well as those with tumors whose NK-activating ligand is associated with worse RFS and/or OS. In addition to the high expression of the specific NK-activating ligand, these tumors of worse prognosis would be more likely to express low levels of NK-inhibitory ligands; thus, reducing the inhibitory signals for NK cell activation and enhancing their cytotoxic potential towards tumor cells. These tumor characteristics might increase the chances for successful NK-based immunotherapy in BC patients by eliminating residual tumor cells, preventing relapse and improving patient survival; thus highlighting the importance of further exploration of the prognostic and therapeutic implications of NK cells in BC in both research and clinical settings.

## MATERIALS AND METHODS

### Identification of the NK-regulatory ligands for NK receptors by literature screening

NK-regulatory receptors (expressed on NK cells) and their respective ligands (expressed on target cells) discovered to date, in humans, were identified by systematic literature screening in Pubmed. First, the keywords “NK” or “natural killer” and “receptor” were used to identify the potential NK receptors and ligands. Then, further literature search was performed, using the keywords “NK” or “natural killer” and “potential NK receptor name” and “potential ligand name” to select all ligands for which articles containing original research data confirming both their interaction with the NK receptor and their regulatory effect on NK activity and cytotoxicity towards the target cell. The exclusion criteria are the ligands that would have been found to bind specific NK receptors without confirmation of their regulatory effect on NK cell activity towards the target cells because these ligands might interfere with the overall conclusions of the study independently of the potential role of NK activity. The selected NK-regulatory ligands for NK receptors are presented in Table 1 and grouped into NK-activating, NK-inhibitory, or NK-activating and inhibitory ligands depending on the biological effect of their interaction with their receptors on NK cells (i.e. activation or inhibition of NK cytotoxic activity towards target cells).

### Analysis of the prognostic values of individual NK receptor ligands in BC patients by using the KM plotter

The correlation between NK receptor ligand members’ mRNA expression and BC patient survival (RFS and OS) was analyzed by KM plotter platform. The analysis included 3955 BC patients for RFS and 1402 BC patients for OS. The BC patients were followed up for 20 years. The prognostic value of each ligand was evaluated either on all the BC tumors or by using several clinical BC criteria and classifications including intrinsic subtypes (luminal A, luminal B, HER2-positive and basal-like) and clinicopathological features (lymph node status, tumor grade, and p53 status). Briefly, individual members of the NK receptor ligands were entered by using their gene symbol into the KM plotter platform. The probe set with the estimated excellent quality (green) was used (the probe set ID for each ligand is indicated in Table 1). “Auto select best cutoff” was chosen in the analysis [158]. Different clinical parameters were selected. Thus, BC samples were split into high and low expression groups according to the cutoff value and the two patient cohorts were compared by Kaplan–Meier survival plots. The hazard ratio (HR) with 95% confidence intervals (CI) and log rank *P* value were calculated then were adjusted using false discovery rate (FDR) for multiple testing correction. All *p* values indicated in the manuscript are FDR-adjusted. HR < 1 implies better survival for high expression group, HR > 1 implies worse survival for high expression group, and HR = 1 implies no effect of ligand mRNA level on survival. The data is considered to be statistically significant when FDR-adjusted *p* value < 0.05.

## Abbreviations

BC: breast cancer
CI: confidence interval
FDR: false discovery rate
EGA: European genome-phenome archive
ER: estrogen receptor
GEO: gene expression omnibus
HER2: human epidermal growth factor receptor 2
HR: hazard ratio
KM: Kaplan–Meier
NK: natural killer
OS: overall survival
PR: progesterone receptor
RFS: relapse-free survival
TCGA: the cancer genome atlas
